# Midwifery care attachments: shaping childbirth agency through care techniques

**DOI:** 10.3389/fgwh.2025.1605546

**Published:** 2025-06-23

**Authors:** Annekatrin Skeide

**Affiliations:** Institute for Midwifery, Charité - Universitätsmedizin Berlin, Berlin, Germany

**Keywords:** midwifery, care, techniques, labor, childbirth, obstetric violence, autonomy, body

## Abstract

Midwifery care has been shown to effectively enhance birth outcomes and improve childbirth experiences. It has, however, not yet been sufficiently articulated how exactly. This study explores how trustful and empowering relationships are crafted through midwifery birthing care techniques. To do so, it builds on insights derived from feminist science and technology studies’ engagements with caring in terms of empirical ethics, namely as situated practices of “doing good”. Using reflexive thematic analysis, I examine semi-structured interviews with midwives alongside ethnographic fieldwork conducted across various midwifery care settings in Germany. Setting two birthing stories in dialogue, I illustrate how bodies-in-labor emerge through collective, active, persistent and adaptive engagements with these dynamic entities in midwifery practice to make physiological childbirth happen. Specifically, I argue that through the midwifery care techniques of “spooning” and “labor and birth positioning” midwifery birthing care attachments are fostered. I conceptualize these attachments as co-responsive, active-passive commitments aimed at sustaining endurable or even pleasurable relationships between embodied selves and bodies-in-labor. Investigating situated midwifery care techniques enables a detailed understanding of their specific qualities in particular childbirth situations, extending conventional notions of being-with and non-intervention. This approach allows to articulate, critically engage with, and strengthen midwifery-specific childbirth care practices.

## 1 Introduction

Midwifery, social scientific and public health research alongside related national and global policies, have recognized that “all is not well with birth” [(1), 4]. Quality maternal healthcare aimed at improving maternal and perinatal health outcomes is unevenly distributed worldwide.^1^ It has been shown that many people giving birth in different environments are systematically threatened, insulted, denied pain medication or coerced into “consent”. The disrespect for and mistreatment of birth givers, which activist groups in Latin American countries have termed “obstetric violence,” has been framed as a global epidemic. Associated with increased maternal morbidity and mortality, obstetric violence is understood as both a public health and a human rights-related issue. It has raised ethico-political concerns about women's health and wellbeing in relation to their autonomy and freedom of choice in childbirth (2–8).^2^

Professional midwifery care has been recognized as the best way to tackle ill-treatment of birth givers, contributing to better health outcomes and positive childbirth experiences (18–21). Midwifery's non-interventionist approach is said to be a humanized counter-program to technocratic, interventionist and obstetric dealings with birthing bodies.^3^ It is argued that midwifery-specific birthing care supports women to cope with birth not only in terms of a physical event but also as a transformative biographical and sociocultural rite of passage. Instead of disciplining and objectifying bodies-in-labor and controlling and alienating women's subjective birthing experiences, midwives foster “normal” physiological births and allow for individual choice and control (22–28).

While the effectiveness of midwifery birthing care techniques in improving labor care has been repeatedly demonstrated, it has not yet been sufficiently articulated, in terms of what they consist of in detail and how exactly they contribute to producing “good” childbirth experiences. In this paper, I draw on insights derived from care studies as a branch of science and technology studies and empirical ethics to demonstrate how midwifery birthing care techniques craft trustful and empowering relationships which involve birth givers as active participants, establishing the conditions for “physiological” births to take place.

## 2 Empirical and theoretical background

Continuity of midwifery care has been shown to provide what matters to “[m]ost healthy childbearing women”: “safety and psychosocial wellbeing” [(29), 2], subsumed under a “positive childbirth experience” [(7, 30); see also (31–33)]. This continuity of care is the provision of “care from the same midwife or team of midwives during pregnancy, birth, and the early parenting period in collaboration with obstetric and specialist teams when required” [(34), 3]. Continuous midwifery care has been shown to foster respectful, woman-centered interactions and to provide safety not only in obstetrical but also in emotional terms (35). In order to demonstrate “the power of midwifery” (19), classic maternal health indicators focusing on life-saving interventions and health outcomes such as maternal mortality [(36), 1750; (13), 2] have been extended to include relational qualities and birth givers' experiences of maternity care. On that basis, two characteristics of maternity care practice have been shown to impact positively on maternal and perinatal health: the timely and indicated use of evidence-based obstetric interventions and respectful and supportive maternity care relationships. The midwifery model of maternity care emphasizes the primacy of the midwife–woman relationship. It equips birth givers, including those from vulnerable and marginalized groups, with a sense of personal achievement. This sense of achievement provides them with an “inner” resource for long-term empowerment (15, 22, 32, 37–43).

A wealth of midwifery research has emphasized the importance of developing trustful and responsive midwifery care relationships over time to care “well” for women giving birth (31, 34, 44–53). Despite their significant impact on perinatal health outcomes and childbirth experience, midwifery care relationships together with techniques aimed at crafting, nurturing and sustaining responsiveness, intimacy and trust have been largely invisible in birth scholarship and birth-related discourses. As opposed to obstetric interventions directed at the body-in-labor which figure in birthing care protocols or guidelines, midwifery birthing care techniques are often attributed to not only a physical but also a psychosocial domain. Evading singularization, standardization and measuring, midwifery techniques which instill “a feeling of trust and safety in a woman who then feels confident to go with the flow of her labour” have “no name”, are “not recorded,” and are “not monitored or accounted for” [(54), 10067]. Traditionally, these techniques have been framed as “expectant management” [(43), 1132], “watching and waiting” [(55), 372], “non-intervention” [(56), 4], or “watchful attendance” (54).

These descriptions of midwifery care techniques have emphasized the relevance of an embodied co-presence of midwives with birth givers. To differentiate them from a medical model of birthing care built on a risk-averse, interventionist approach, these framings have foregrounded a more receptive “non-doing”, also suggesting that there is not much happening to talk or to write about. Ideally rather passive and thus unobtrusive (non-/low-interventionist) midwifery care practices have also been argued to foster birth givers' position as active and competent choosers. The position of “‘consumers’ making choices about birth” has been idealized since the 1970s, especially in contexts located within the Global North and associated with the middle classes [(1), 40]. Care ethical approaches have been introduced by midwifery scholars as a radically relational alternative to positioning birthing women as autonomous agents (57–61). Midwifery scholars have also argued for better articulation of specific midwifery birthing care techniques beyond just watching and waiting. In addition to focusing on what midwives do *not* do, or do *less* of, namely using obstetric interventions, more studies are needed on what midwives do when attending births (including using obstetric interventions), how exactly they do it, and with what effects (54).

In this paper I aim to contribute to these discussions on how to conceive of and to talk about midwifery care practices. My inspiration comes from feminist science and technology studies' (STS) engagements with caring in terms of empirical ethics, namely as situated practices of “doing good” (62–65). In this tradition, care as a practice is not confined to healthcare alone, even though healthcare studies—particularly nursing theory—were instrumental in drawing academic attention to care as a set of central socio-material activities that shape and constitute daily life (65). Sensitivities informed by Science and Technology Studies (STS) have contributed to decentering human actors and agency by focusing on the relationships between people, technologies, environments, and words. The identities of people, bodies, or things cannot be predefined once and for all; rather, they emerge as effects of the relationships they establish within specific care practices. This approach posits that “everything in the social and natural worlds [is] a continuously generated effect of the webs of relations within which they are located” [(66), 141]. It assumes a “radical relationality” [(67); see also (60)] that serves as both a methodological framework and a methodical tool. This radical relationality also extends to the ways in which care practices are studied and understood in and through research. Researchers, too, establish and cultivate specific relationships with their research “objects.” Within this scholarship, research practices are understood as “re-scriptive”: the questions researchers ask and the methods they use to answer them actively shape and bring specific research objects into being, rather than “discovering” them as pre-existing or given [(67), 179]. Against this backdrop, it has been emphasized that “if care studies are not carefully attended to, there is a risk that they will be eroded” [(65), 7]. Consequently, research in this field often investigates specific, local care practices—typically through ethnographic or, more precisely, praxiographic methods (68)—with the aim of “improving care in its own terms” [(69), 2]. Both care practices and research practices are inherently normative, as they are “oriented towards achieving something good.” Describing—or re-scribing—care practices in terms of empirical ethics involves attending to the “goods,” the norms, and the values that midwives, birth givers, or birthing environments implicitly or explicitly strive for or mobilize, as well as “the ‘bads’ they want to avoid” [(67), 177].

Informed by this approach, I investigate midwifery care as an embodied practice that contributes to restoring, sustaining, or improving birthing situations [(70), 185]. I illustrate how, in birthing care arrangements that strive for “giving birth” well, entities such as bodies-in-labor are brought into being or “enacted” in various ways. For example, they may become both corporeal actors *and* objects of different interventions and assessments (71).

To develop an empirically grounded vocabulary for the specific appreciative and creative forms of midwifery birthing care relationships, I draw on a wide array of literature^4^ that shares the theoretical commitments described above. These studies challenge conventional notions of agency as a human property confined within individuals and shaped by external structures, as subjectivity in opposition to objectification, or as activity contrasted with passivity. Instead, they use empirical material to generate relevant and often surprising insights that not only deepen our understanding of the practices being described but also contribute to theoretical developments on selfhood, embodiment, and ethics.

Against that background, (that has not yet been received within midwifery research) I propose a radically relational understanding of midwifery care practices. My contribution introduces a relational and distributed notion of birth givers' agency, emphasizing how agency emerges through the dynamic interplay of birthing care relationships, practices, and environments. To do this, I address the following research questions: How are birthing care relationships configured in midwifery care practices? Which modes of giving birth “well” are enacted through midwifery birthing care techniques?

## 3 Methods and material

The ethnographic material used in this paper originates from Germany. In Germany, pregnancy, birth, and postpartum care are typically fragmented, occurring in various settings and involving multiple obstetricians and midwives. The common pathway through the contemporary landscape of German maternity healthcare begins with monthly, and later biweekly, prenatal care provided by obstetricians in their practices. This is followed by prenatal classes taught by midwives in designated facilities. Birth takes place in a clinical labor ward, attended by both midwives and medical doctors, with the latter being in charge. During the subsequent days, nurses, midwives, and medical doctors care for women and newborns in the maternity ward. From the third day postpartum, a midwife conducts home visits until twelve weeks after birth. Six weeks after delivery, the woman and child return to the obstetrician for a follow-up examination. I utilize material gathered from both Eastern and Western Germany, as historical, structural, and societal differences have led to variations in the organization of maternity care and working conditions. In the former German Democratic Republic (GDR, 1949–1990), maternity care was centrally and state-organized, with out-of-hospital births being virtually nonexistent, at least officially. In contrast, the Federal Republic of Germany (FRG) has seen a greater variety of care models, including midwife-led birthing centers [Geburtshäuser]. The data supporting my arguments include eleven semi-structured interviews conducted in 2022 and 2023 with midwives working in hospitals, private homes, midwife-led birthing centers and ob-gyn practices in the eastern states of Germany.^5^ The aim of these interviews was to gain a deeper understanding of how midwives facilitate, maintain, and restore physiological birth, as well as to clarify what constitutes physiological birth under various conditions. To achieve this, we employed a purposive sampling approach, selecting midwives who have worked in both clinical and extra-clinical environments for at least five years and who chose to become midwives more out of a sense of calling than merely as a job [(76), 93].

In addition, I used ethnographic material from the fieldwork I conducted as a PhD student between February 2015 and March 2016 in var­ious sites where midwives work in northern and eastern Germany, including six observational protocols, eight formal interviews and various informal conversations with midwives and women.^6^ In this project I addressed the following research questions: What are midwifery care techniques? What do bodies become in midwifery care arrangements? How can “good” midwifery care practices get strengthened in and through research?

In both research projects, several ethical considerations were meticulously addressed to ensure the well-being of participants and maintain the integrity of the research process. Prior to participation, all individuals were provided with a detailed written and verbal explanation of the respective project, including its procedures and objectives. This approach created transparency and fostered trust between the researcher and the participants. To further protect participants' identities and safeguard their privacy, anonymization and pseudonymization of the data were implemented throughout the study. Informed consent was obtained from all participants regarding their involvement in the research. Participants received a consent form that clearly stated their participation was voluntary and could be withdrawn at any time without any negative consequences. Additionally, I signed a confidentiality agreement to ensure that all information collected during the research would be treated with confidentiality. These ethical measures were crucial in upholding the standards of the research and strengthening participants' trust in the project, thereby ensuring a responsible and ethical approach to data collection and analysis.In order to analyze the data set, I used a reflexive thematic analysis (reflexive TA) approach (80–82) with the aim of developing sensitizing concepts as pointers to “suggest directions along which to look” [(83), 7]. As an alternative to presenting a coherent explanatory theoretical framework that can be “applied”, for example, to evaluate midwifery care practices “in general”, I seek for contributing to further refining the theoretical concepts that address the qualities and effects of particular midwifery care practices in order to strengthen and to improve them.

In the results section, in which I theorize midwifery care attachments, I introduce the conceptual themes through excerpts from two interviews which form part of the above-mentioned data set. One of the interviews was with a midwife I will call Madeleine^7^, who was working in a midwife-led birth center. The other interview was with Saira who had given birth in a hospital. I set Madeleine's and Saira's quotes in dialog to study midwifery care attachments and their techniques *across* environments (hospital and community), models of care (medical and midwifery), genres of knowledge (clinical expertise and “patient” experience) or perspectives (midwife and birth giver).

In keeping with ethnographic practices that prioritize depth and nuance over breadth, I present two cases from a larger dataset comprising 19 interviews, eight observational protocols, and informal conversations. The selection of these two cases is informed by a comprehensive analysis of the entire dataset. This ensures that the cases exemplify the themes identified during the analysis of the full dataset. This approach allows for providing rich, detailed narratives that illuminate the complexities of relationships within midwifery birthing care over time. I have also chosen to set these two cases in dialogue specifically to challenge common juxtapositions often found in discussions of midwifery birthing care. These include (a) the perspectives of the midwife vs. the birth giver, (b) the birthing care environments (midwife-led out-of-hospital settings vs. hospital-based contexts), and (c) the birthing care models (midwifery model of care vs. medical model of care). By examining these cases in conversation with one another, I aim to complicate and move beyond these traditional binaries, offering a more nuanced understanding of midwifery practice and the dynamics of birthing care relationships.

When presenting the results, I draw on literature inspired by Science and Technology Studies (STS) that (re)conceptualizes agency. I cite this literature not only to acknowledge its influence on my thinking but also to emphasize the importance of reflexivity in my research process, including its outcomes. Ethnographic results are not merely raw data; they are interpretive, and literature citations help to make this interpretive nature visible.

## 4 Results

The interview with midwife Madeleine was held in 2022. Madeleine had worked as a nursery teacher for nearly twenty years before she decided to become a midwife. After the training she started to work on a labor ward at a university hospital. She resigned after a year because she did not appreciate what she described as a rule-based approach, which applied “a specific perspective even though that perspective doesn't apply to everyone,” and which followed rules “just because that's the way it always has been.” Madeleine instead decided to work at a midwife-led birth center, where she has been working together with six midwives organized in teams for the last eight years. Madeleine and her colleagues provide prenatal, birthing and postpartum care and accompany births both at the birth center and in people's homes. In her work, Madeleine feels that “the situation is more important than the rule” and care is provided based on “a good overall view.”

The second interview and set of quotes I use stem from an interview I held with Saira in 2015. Accompanied by her husband, Saira had given birth to her first child in the hospital where I was doing my ethnographic fieldwork. Saira agreed that I could observationally participate at her birth and conduct an interview with her two days later.

### 4.1 Embodying labor

Asked how exactly she promotes normal, physiological births in her work, Madeleine describes a “memorable birth attendance [eindrückliche Geburtsbegleitung]” in the birth center:

Last year I accompanied Lisa and her partner Ole in the birthing place. Lisa was a first-time mother with a completely normal prolonged labor. ((laughing)) That makes you laugh too. Because “prolonged” is technically no longer physiological. ((laughing)) Exactly. So it was actually just a normal, lengthy accompaniment for a first baby. And eventually, of course, the couple were exhausted. And I was also a bit tired because we had been pretty busy here. I think it was the third birth in two days. And then Lisa and Ole called me again and said, “This isn’t working at all. The contractions are terrible [furchtbar]. We’re considering a transfer to the hospital. We can‘t go on like this. We need an epidural.”

The interviewer Kristin's laughter is nourished by her own experiences of attending hundreds of “normal prolonged labors” in out-of-hospital settings for over thirty years. Both Kristin and Madeleine know that in practice, each labor has its proper dynamics. The duration and “progress” of labor vary considerably while also being reliantly “lengthy […] for a first baby”, as Madeleine puts it. Madeleine assesses the situation as a “completely normal prolonged labor” for a “first-time mother” giving birth in the birth center. Just like many times before, the further course of the labor provides evidence for her evaluation: Lisa gives birth to a healthy child without any complications about one and a half hours later. However, in the scene described by Madeleine, Lisa and Ole felt stuck. They were overwhelmed by the length and exhaustion of Lisa's labor. She had been subjected to “terrible” contractions for hours, leaving Ole desperate and eager to help, yet helpless. There seemed to no end in sight as there were few signs that the labor was “progressing” towards that end. Such signs of labor progress could consist of Lisa feeling a different quality, intensity and direction of pressure, of Madeleine palpating a further opening of the cervix, or of Madeleine not leaving the room as a sign that birth is now imminent. As none of this had occurred, Ole and Lisa were left unrewarded and with “terrible contractions.” How Lisa's body-in-labor is assessed, be it through physical self-awareness, through obstetric intervention or through the midwife's attentive co-presence, validates or disproves Ole's and Lisa's strategies to handle that body. Also in this out-of-hospital environment, Lisa's body-in-labor is “constituted through extremely varied mediations, among which obstetrical expertise plays a significant role” [(71), 66]. The labor process emerging as “prolonged” renders Ole's and Lisa's labor strategies ineffective and Lisa's body-in-labor inaccessible and expendable. At this point, the efforts they have invested into laboring seem to be in vain. This enactment of her body-in-labor strips Lisa of her agency, nourishing her wish to distance herself further from or to even “get rid” of her body-in-labor, by escaping its “terrible” contractions via an epidural, which would then also be a strategy to regain agency.

Saira's childbirth takes place under different conditions. Saira's ob-gyn referred her to the hospital three weeks before her due date, suggesting that labor may need to be induced. After four days in the maternity ward on misoprostol treatment to induce labor, Saira was finally admitted to the labor ward where she spent another twenty hours, walking, bathing, lying, sitting, and, eventually, “doing a circus there”, as she described it, adopting various uncomfortable birthing positions to facilitate birth. Saira explains:

I did not demand anything [hatte keine Ansprüche an irgendwas]. I just wanted to bring a healthy child into the world and preferably by my own strength. Because my diagnosis was macrosomia. That means, the child could be bigger than the mother could tolerate, and it could lead to complications. And my wish was to not get a c-section. That's why I was induced. But I reached a point at which I thought: “Okay, this is it. I can’t do this anymore.” It was progressing, but everything was sooo slow, you know. They [the midwives and obstetricians, A.S.] said: “We’re pretty much on track with the birth. It can drag on, especially with the first child and the induction and all.”

The midwives working on the labor ward were skeptical about both the accuracy of the diagnosis, fetal macrosomia, and the resulting intervention, the induction of childbirth, prescribed by their medical colleagues in the hospital. The midwives argued that the sonographic measurements on which such a diagnosis is based are often imprecise and that the fetus did not feel overly big when they palpated Saira's belly. They also pointed out that the cesarean section Saira wanted to avoid had been a common result of attempted labor inductions on their ward. When talking to me or to their medical colleagues, the midwives made clear that they would have favored an expectant management. However, they did not share their skepticism and preferences with Saira. That was because Saira, for her part, felt relieved that something was being done. Accepting “her diagnosis” as an indisputable fact, she shares the goal the induction is aimed at: to avoid the dangers arising from that diagnosis and to work with its challenges, and to bring “a healthy child into the world and preferably by [her] own strength.” Saira aligns her interests with those suggested by the obstetrical definitions and procedures in the hospital. This requires her active engagement in guiding and managing her body-in-labor, as Saira explains in more detail below. Being exhausted makes fragile the subject position enacted by and for Saira, marked by a sovereign distance towards her body-in-labor. A sovereign distance towards her body-in-labor maintains Saira's connection and authority, rather than creating alienation and disconnection. Saira's body-in-labor is, however, not at her exclusive disposition and her sole responsibility; her relational agency is distributed over obstetric procedures, technologies, practitioners and the medical setting with which Saira shares responsibilities and activities directed at her body-in-labor.

Obstetrical knowledge, instruments and gestures are rarely explicated and marked as such in the birth center, but rather incorporated into conversations and interactions. This is different in the hospital, tasked with and relying on monitoring, diagnosing, and treating. Nevertheless, obstetrical descriptions are crucial for both Saira's and Lisa's bodies-in-labor to emerge as acting corporeal entities in their own right [(71), 81]. Saira feeling that she “can't do it anymore,” Lisa and Ole stating that they “can't go on like that,” provide turning points for re-evaluating situations in which their bodies-in-labor stubbornly do not live up to the ideals of a “clockwork birth,” a “‘textbook’ medical version of ‘normal birth’,” [(1), 52] – despite the efforts invested into aligning both. These embodiments challenge Saira's and Lisa's “integrities” as embodied selves: their bodies-in-labor seem dissociated and inaccessible yet powerful, potentially overwhelming actors [(71), 73]. Imposing themselves upon Saira and Lisa, their bodies-in-labor make them react but seem to resist their labor strategies. Midwifery birthing care techniques aimed at developing alternatives to these threatening constellations are oriented towards embodied selves and bodies-in-labor “getting in sync”, as I discuss below.

### 4.2 Making birthing happen

Madeleine continues:

I said I thought it would be great if Lisa could get some rest at this point. She immediately responded, “I can’t sleep, it’s just not possible.” And I replied, “Okay, here’s the plan: you’re going to try to rest one more time. We’ll make it dark here, help you get into it.” And that worked for about 20 min ((laughter)) – well, it didn't really work because I could hear during the contractions that she was jumping up again. I went back into the birthing room. By then, Lisa had become really, really hysterical: “This is just impossible! I can’t take it anymore! I don’t have any breaks at all!” And she really didn't have any breaks between contractions – it was one after another. Ole was also completely desperate because he couldn't help her. We had a quick discussion, and I sent him to sleep, telling Lisa, “You know what? What you need now is someone to breathe with you and to get actively involved. We need calm now. You can feel it yourself – you’re completely overwhelmed and don’t have control over what’s happening anymore.

When Lisa and Ole state: “We need an epidural,” Madeleine could have responded to this request. Backed up by the irrefutable truth of Lisa's labor pains, her physical and emotional exhaustion and by the indication of “prolonged labor,” a transfer to the hospital might have seemed a reasonable way to “go forward”. No one would have been surprised: in Germany, prolonged or obstructed labor and maternal requests for extended pain management are the two most frequent reasons for a transfer from home or a birthing place to the hospital during the first stage of labor [(84), 41].

But Madeleine tells a different story: as they prepared for Lisa's birth in the birth center together with Madeleine during the last months, Lisa and Ole also had a longer conversation with her about possible scenarios involving a transfer to the hospital. Such a transfer emerged as a last resort, necessary and urgent in case of emergency situations which are rare. In the course of Lisa's pregnancy, Lisa, Ole and Madeleine prepared well to make giving birth in this environment work. To prepare, they had become acquainted with each other *in this environment*, the birth center. Lisa and Ole also attended yoga classes and birth preparation courses, read through blogs, forums and books, or chatted with friends, parents and strangers in order to learn a vast repertoire of practical labor knowledge. Madeleine interpreted Lisa and Ole “considering a transfer to the hospital” as them needing support in order to continue laboring in the birth center. As a first supportive mediation, Madeleine prepares the room for inviting Lisa and Ole “to try to rest one more time.” Lisa and her body-in-labour are indeed affected by the dark and calm surroundings, however, not in the way Madeleine had intended, nor how Lisa and Ole had hoped for. Lisa gets “completely overwhelmed” by her body-in-labor responding with more frequent and unbearable contractions.

Madeleine observes that at that point, Lisa does not “have control over what is happening [Kontrolle über das Geschehen].” What exactly does “control over what is happening” during Lisa's labor, which Madeleine refers to, encompass? “Being in control” is a dominant ideal in scientific, policy-related and activist childbirth discourses. However, giving birth complicates classic understandings of human agency as Madeleine's as well as Saira's birthing stories show: to give birth is neither “an external power that forces itself upon” Lisa and Saira as passive and manipulated subjects, but nor is it purely the result of Lisa's and Saira's capacities to act as willful subjects. Birthing (significantly called) spontaneously “just happens” *and* is prepared to happen in specific ways [(73), 112]. It is thus not possible to be fully prepared for birthing's unavoidable ’spontaneity'. But it is possible to approach unpredictable bodies-in-labor in ways that render their handling easier and more enjoyable. The birthing care technique mobilized in response to Lisa's exhaustion is an engagement in creating the conditions for improving the birthing situation (69). This is done through actively, perseveringly and adaptively *working with* the body-in-labor.

Saira emphasizes how strenuous the work necessary to make spontaneous childbirth happen for her was:

It had to be stimulated even more, the baby had to be in a position so that it could slide through the birth canal, and it didn't really want to, and I had to go along with it exactly in order to reach my goal. I had to take on such strange positions! I really had to do a circus there! If I had just laid there completely calmly and acted like in a movie: pressed three times and the baby is there – that would never have worked. I really had to go through everything. And I did it blindly. I just functioned. I would now say the mind was switched off, and I just did what I was told because I trusted and knew I was in good hands. And in the end, the baby would come.

Saira, who had taken part in a labor and delivery tour in the hospital and in a birthing preparatory course to prepare for giving birth in the hospital, describes the many hours preceding the actual birth of “the baby” filled with active, arduous and painful birthing work, but with the labor usually being omitted in fictional birthing scenes in movies. “Behind the scenes”, Saira's body-in-labor is worked upon and with to make giving birth happen. Equipped with a walking epidural, guided and supported by the midwife accompanying Saira during the last hours of labor, Saira has “to do a circus,” exerting a vast repertoire of “strange” labor positions to, eventually, give birth. In the midwifery birthing care technique of labor and birth positioning, Saira's body-in-labor is turned into as an instrument and object of giving birth in the positional techniques Saira describes. Objectifying and instrumentalizing Saira's body-in-labor by using these birthing care techniques could result in a dissociation between embodied self and body-in-labor. These dissociative relationships have been described as (at least potentially) alienating (85–89). However, as Saira's story demonstrates, that is not necessarily the case. Saira “go[es] along with” working with her body-in-labor “under the authority and expertise of others” [(75), 567], in order to achieve “her goals”. Her strategy of actively subordinating herself to objectifying procedures and strategies in order to realize her goal “to bring a healthy child into the world” by her “own strength” is a way to exercise agency [(75), 595]. Saira engages active-passively in being guided and in realizing the instructions for “strange” and potentially shameful labor positions. As mentioned before, the interventions and activities directed at Saira's body-in-labor, together with their accountabilities, are distributed over a heterogenous birthing care collective, involving healthcare staff, obstetrical technologies and standards, or the clinical labor ward. As part of this collective, Saira is allowed “just to be functioning”, which is to actively participate in making birth happen by following external guidance. Sharing accountabilities does not only facilitate Saira to “go through everything” but is also a condition for *building* trust, for Saira to *become familiar with* being “in good hands.”

### 4.3 Creating birthing care attachments

Creating a calm environment did not help to improve Ole's and Lisa's situation. Lisa needed someone “to get actively involved”, as midwife Madeleine explains further:

Then I actually lay down with Lisa in a spooning position on the bed. We held each other tightly, and I breathed through every contraction with her. Suddenly, small breaks [in-between contractions] started to appear. And I think the warmth at her back and the calm, active participation helped her find her footing again [zur Ruhe zu finden]. Before we lay down, Lisa was at three centimeters, and three-quarters of an hour later, she started pushing ((laughter)) and was suddenly fully dilated. It was such a striking moment! There was absolutely no indication that a transfer [to the hospital] was needed, except that they were just completely exhausted and had no strength left. And, of course, I could have just said, “I’ll step out for a bit – you can handle this.” But then, I probably would have ended up transferring her at some point.

Lying together on the bed, holding each other tightly and breathing together is part of a repertoire of midwifery care techniques specific to out-of-hospital birth attendances. The spooning Madeleine describes is enabled by and illuminates what characterizes midwifery care practices in these sites: a comfortable bed big enough for hosting two people instead of a delivery table, the absence of hospital hygiene rules prohibiting close bodily proximities, but the surrounding's invitation to come close to each other, the continuous co-presence of a familiar midwife in an equally familiar, undisturbed environment, and a trustful relationship crafted through the continuity of care of prenatal encounters, which were occasions for getting to know each other. “Spooning”, a midwifery birthing care technique of “being-with” (47) or “working-with” (46) seeks, I argue, to foster *attachments*. Cultivating attachments is a collective endeavor distributed over several different agents, including Lisa, the homely atmosphere, Madeleine's “warmth at her back and the calm, active participation,” and the cozy bed. Gathered together with Lisa, they invite her to engage with her body-in-labor through “trust and interest” [(72), 115], to let herself be moved and affected by her body-in-labor but also to effectively move and affect her body-in-labor [(72), 113]. Through collectively embodying trust and interest, attachments are formed which help Lisa “to re-incorporate” her body-in-labor. These relationships are “a strange mixture of active and passive” [(74), 12]: in order to come to rest, Lisa holds Madeleine and breathes with her, she makes herself available to the invitation of her midwife and her surroundings to “find her footing again”. But Lisa is also being held and breathed with and invited to respond to the offers. Through spooning Lisa co-guides and co-manages her body-in-labor *in order to* let go of striving for complete control of her apprehensions and management of her labor, and to avoid a complete loss of control. As Madeleine describes, this technique helps Lisa to “find her footing again”; her body-in-labor responds with “small breaks” in between the contractions. Lisa starts to push “three-quarters of an hour later”. Lisa's appreciation of holding and being held by Madeleine and of synchronizing their breathing is an embodied enactment of “safety and psychosocial wellbeing” [(29), 2; see also (90)], as Madeleine's description above suggests.

The midwifery birthing care technique of “labor and birth positioning” mobilized in the hospital under clinical conditions shows more similarities with the ’spooning' seen in the birth center than it may seem at first sight. Saira continues:

“And, yes, I realized that she [the midwife] was really interested in finally reaching the goal. She really wanted to take this burden off me. She wanted to get through this birth with me. And if it hadn't been for her, if she hadn’t been the one to push me so much yesterday, I don’t know what would have happened. She said that I was being really brave. And I saw in her eyes that she meant it seriously. Not that standard: ‘You’ve got this,’ which she has to say to every woman. I saw it, that she thought: ‘Wow, this one is really strong.’ I saw it in her eyes. And, you know what, she even thanked me for this beautiful birth.”

Just as Madeleine had done, Saira's midwife had prepared an environment suitable for the exigencies of “doing circus”, installing a mattress, a gymnastics ball as well as cushions in order to provide support for Saira to take “strange positions.” Saira's midwife is also co-present to engage in working with Saira's body-in-labor through demonstrating body postures, massaging, or, as Saira emphasizes, through motivating and “pushing”. Both techniques, “spooning” and “labor and birth positioning”, are aimed at forging attachments, trustful and interested relationships, expecting that the efforts invested in handling bodies-in-labor are going to be successful and responsive to a body-in-labor's idiosyncrasies and exigencies. Just like Lisa, Saira makes herself available to “the expectations of someone who cares, of someone who trusts, moreover, of someone who was interested, someone it interests” [(72), 124]. Saira emphasizes the importance of her deep engagement, efforts and success being validated by the midwife – while the latter hid her own contribution. According to her midwife, the “circus” Saira “had to do” was a “beautiful birth.” This aesthetic qualification is important for preventing Saira becoming alienated or even traumatized, as it values Saira's strenuous and creative efforts in making birth happen “spontaneously” (and avoiding a c-section). Saira and her midwife's goals of “giving birth to a healthy child by one's own strength” were aligned, as were those of Lisa and Madeleine, which facilitates the creation and cultivation of attachments, allowing combined efforts for working with Lisa's and Saira's bodies-in-labor.

Both birthing trajectories ended well, rewarding the efforts invested. Saira sets these efforts in a causal relationship to the outcome, retrospectively validating her investments, guided and supported by her midwife, to make birth happen. Madeleine is more hesitant to do so. She describes Lisa's birth as a “remarkable” case because she knows that midwifery care techniques such as “spooning” or “labor and birth positioning” may also fail – even if birth givers engage as responsively as Lisa and Saira. These techniques are both, adaptions to and explorations of continuously evolving labor situations. However, even if the techniques I presented would have failed to eventually make “spontaneous” birth happen, they create empowering attachments as conditions for giving birth “well”.

## 5 Discussion

I have described midwifery birthing care relationships that go beyond the often focused-upon dyadic relationship between women and midwives, understood as midwifery's primary ethical relationship. Giving birth happens and is made to happen through social and material midwifery care collectives of not only birth givers, their companions, midwives and medical doctors, but also birthing care surroundings and their material and emotional affordances, their standards, goals and ideals. Two care collectives were analyzed in this paper: one situated in a midwife-led birth center and the other in a hospital. In these care collectives, bodies-in-labor are configured as central actors also through obstetrical articulations. That means that in both environments how bodies-in-labor are and can be inhabited is also mediated by obstetrical descriptions and interventions. Through the midwifery care techniques of “spooning” and “labor and birth positioning” Lisa and Saira are invited to actively participate in working with their bodies-in-labor. They learn to become sensitive to their bodies-in-labor in ways that allow them to affect their bodies-in-labor instead of being overwhelmed or alienated. These techniques aim at cultivating midwifery birthing care attachments that I understand as collective, co-responsive, active-passive commitments aimed at sustaining endurable or even pleasurable relationships between embodied selves and bodies-in-labor. These midwifery care attachments are brought about through highly organized activities – and passivities – extending co-presence or non-intervention.

I argued that instead of the midwives striving for Lisa and Saira to control themselves, their bodies-in-labor and what happens to them or to surrender to their bodies and the events, Lisa and Saira are caringly invited to engage with their bodies-in-labor, trustfully and interestedly, in order to give birth “spontaneously” in both the out-of-hospital and the medical environment. Lisa's and Saira's capacity to act is distributed over and mediated by various other actors or participants which are interrelated and interdependent: their midwives, the birthing care surroundings, obstetrical definitions and procedures, even motivational words and caresses. In practice, their positions stand thus in stark contrast with consumerist agendas presupposing liberal subjects being in control and making choices. Promoting a “logic of care” instead of a logic of choice and control (69) makes it possible to articulate the collective creative techniques mobilized in midwifery care practices. These midwifery birthing care techniques grapple relentlessly and adaptively with more-than-medical uncertainties and fragilities as part of giving birth. These techniques act speculatively upon what might be “good” for this particular person and body-in-labor in their particular circumstances, without any participant, however, knowing *for sure* what exactly this “good” might entail.

Being “with woman” is not just an ideal or ethical obligation, especially important in continuous midwifery care constellations, but a laborious, shared and hands-on endeavour. It necessitates cultivating particular responsivities towards the offerings of the environment, the midwifery care relationship or the body-in-labor in order to make giving birth work (48, 52, 91). By understanding and articulating the relationalities of physiological birth through midwifery birthing care attachments, as situated, dynamic and collective endeavors, we may also present alternatives to two challenging trends in birthing: the relentless expansion of repertoires of risk avoidance because of the unpredictability of giving birth; and the “blame culture” associated with these strategies, pinning adverse outcomes systematically down to wrong decisions made by individuals, be it birth attendants or birth givers [(92), 209].

While my findings are specific to the environments in which the events occurred, they can still be relevant in other contexts. Instead of generalizing the insights presented here, they can be utilized as a methodological and conceptual lens for exploring midwifery care techniques that foster supportive attachments, such as “spooning” or “labor and birth positioning”, in various settings and times.

## 6 Conclusion

Obstetric violence has often been framed as a public health and human rights-related concern but less attention has been given to studying the concrete social and material conditions through which birth givers “can claim and recognise selfhood in their actions” [(93), 34]. While depictions of obstetric violence and humane counterprograms do not leave much space to examine the more nuanced and “broad spectrum that lies between complete lack of connection, on the one hand, and actual “intersubjectivity,” on the other hand” [(94), 244], my suggestion is to lay open and analyze that space through studying midwifery care relationships *in practice*. This approach helps to carve out surprising and important nuances. While one might assume that obstetric violence “has much in common with the more general experience of alienation and objectification within medicalization” [(95), 241], with alienation being “at the kernel of birth trauma narratives” [(96), 496], my investigation of concrete and situated midwifery care practices shows that birth givers may actively take part in objectifying their body-in-labor in order to “reach their goals”, thereby exercising agency and avoiding alienation. Approaching agency in childbirths through a logic of care instead of a logic of control allows acknowledgement of “interdependency as the ontological state in which humans and countless other beings unavoidably live” [(97), 4] and an avoidance of “maternal separation” (60).

In one of the cases presented in this paper, I have demonstrated that decisions not (yet) to give pain relief cannot be necessarily understood as a “failure to meet professional standards of care” [(2), 11] but may also constitute an act of caring. My analysis suggests that defining what “bad” or “good” maternity care consists of in terms of singular (non-)interventions may not be sufficient. The concrete and particular socio-material contexts have to be considered to understand better how “goods” and “bads” in maternity care are constituted.

Investigating situated midwifery care techniques allows to capture the specific and detailed qualities of what is done in particular childbirth situations. Thus, this contribution demonstrates how “investigating local minutiae might actually be crucial to provide general insight” [(98), 158]. This facilitates tracing midwifery care relationships as “the hidden threads in the tapestry of maternity care” (44), so they can be seen, meaningfully engaged with and further strengthened.

## Data Availability

The raw data supporting the conclusions of this article will be made available by the authors, without undue reservation.

## Footnotes

^1^Inequalities exist between scarcely resourced and well-equipped maternity care environments, with structural, personnel-related and access-related differences. Disparities have also been described across socio-political and demographic categories, such as race, ethnicity, or socio-economic background (99–101).

^2^Framed as “a specific form of violence against women” [(9), 61; see also (10, 11)], obstetric violence affects certain groups of women and people more frequently than others. These are women and people assigned to a different, non-majority identity, relating for example to class, ethnicity, race, religion or age, as well as women giving birth in a hospital (5, 12–17).

^3^Not using particular obstetric interventions such as epidurals or cesarean sections while using others such as fetal heartbeat monitoring is anchored in midwives’ legally defined scope of practice.

^4^In this paper, I use feminist STS inspired research on veterinary, ethological, excreting, amateur but also reproductive medical and maternity care practices (64, 71–75).

^5^I thank Kristin Rammel, Diana Briel and Bettina Staudenmeyer for conducting the interviews.

^6^The following publications document my PhD project: (48, 77–79).

^7^All names used are pseudonyms to protect anonymity.

## References

[1] Chadwick R. Bodies That Birth: Vitalizing Birth Politics. Women and Psychology. London, New York: Routledge (2018).

[2] Bohren MA Vogel JP Hunter EC Lutsiv O Makh SK Souza JP The mistreatment of women during childbirth in health facilities globally: a mixed-methods systematic review. PLoS Med. (2015) 12(6):e1001847; discussion e1001847-e1001847. 10.1371/journal.pmed.1001847 26126110 PMC4488322

[3] Bowser D Hill K. Exploring Evidence for Disrespect and Abuse in Facility-Based Childbirth: Report of a Landscape Analysis. USAID- TRAction Project, Harvard School of Public Health (2010).

[4] Jardim DMB Modena CM. Obstetric violence in the daily routine of care and its characteristics. Rev Lat Am Enfermagem. (2018) 26(e):3069. 10.1590/1518-8345.2450.3069 PMC6280177 30517571

[5] Miller S Lalonde A. The global epidemic of abuse and disrespect during childbirth: history, evidence, interventions, and FIGO’s mother−baby friendly birthing facilities initiative. Int J Gynecol Obstet. (2015) 131(S1):S49–52. 10.1016/j.ijgo.2015.02.005 26433506

[6] Shabot SC. We birth with others: towards a beauvoirian understanding of obstetric violence. Eur J Womens Stud. (2021) 28(2):213–28. 10.1177/1350506820919474

[7] WHO. WHO Recommendations. Intrapartum Care for a Positive Childbirth Experience (2018). Available at: https://www.who.int/publications/i/item/9789241550215 (Accessed December 12, 2024). 30070803

[8] van der Waal R. Birth Justice: From Obstetric Violence to Abolitionist Care. Amsterdam: Amsterdam University Press (2024). 10.5117/9789048562398

[9] Murray de Lopez J. When the scars begin to heal: narratives of obstetric violence in Chiapas, Mexico. Clin Gov. (2018) 23(1):60–9. 10.1108/IJHG-05-2017-0022

[10] Berzon C Shabot SC. Obstetric violence and vulnerability: a bioethical approach. IJFAB. (2023) 16(1):52–76. 10.3138/ijfab-16.2.02

[11] Sadler M Santos MJ Ruiz-Berdún D Rojas GL Skoko E Gillen P Moving beyond disrespect and abuse: addressing the structural dimensions of obstetric violence. Reprod Health Matters. (2016) 24(47):47–55. 10.1016/j.rhm.2016.04.002 27578338

[12] Vedam S Stoll K Taiwo TK Rubashkin N Cheyney M Strauss N The giving voice to mothers study: inequity and mistreatment during pregnancy and childbirth in the United States. Reprod Health. (2019) 16(1):1–18. 10.1186/s12978-019-0729-2 31182118 PMC6558766

[13] Bohren MA Oladapo OT Tunçalp Ö Wendland M Vogel JP Tikkanen M Formative research and development of innovative tools for ‘better outcomes in labour difficulty’ (BOLD): study protocol. Reprod Health. (2015) 12(1):50. 10.1186/s12978-015-0028-5 26006320 PMC4464989

[14] Fannin M. Labour pain, ‘natal politics’ and reproductive justice for black birth givers. Body Soc. (2019) 25(3):22–48. 10.1177/1357034X19856429

[15] MacLellan J Collins S Myatt M Pope C Knighton W Rai T. Black, Asian and minority ethnic women’s experiences of maternity services in the UK: a qualitative evidence synthesis. J Adv Nurs. (2022) 78(7):2175–90. 10.1111/jan.15233 35332568 PMC9314829

[16] McCalman P Forster D Newton M McLardie-Hore F McLachlan H. ‘Safe, connected, supported in a complex system.’ Exploring the views of women who had a first nations baby at one of three maternity services offering culturally tailored continuity of midwife care in Victoria, Australia. A qualitative analysis of free-text survey responses. Women Birth. (2024) 37(3):101583. 10.1016/j.wombi.2024.01.009 38302389

[17] Klittmark S Malmquist A Karlsson G Ulfsdotter A Grundström H Nieminen K. When complications arise during birth: LBTQ people’s experiences of care. Midwifery. (2023) 121:103649. 10.1016/j.midw.2023.103649 37003045

[18] Nove A Friberg IK de Bernis L McConville F Moran AC Najjemba M Potential impact of midwives in preventing and reducing maternal and neonatal mortality and stillbirths: a lives saved tool modelling study. Lancet Glob Health. (2021) 9(1):e24–32. 10.1016/S2214-109X(20)30397-1 33275948 PMC7758876

[19] Horton R Astudillo O. The power of midwifery. Lancet. (2014) 384(9948):1075–76. 10.1016/S0140-6736(14)60855-2 24965820

[20] WHO. WHO Statement. The Prevention and Elimination of Disrespect and Abuse during Facility Based Childbirth. (2015). Available at: https://iris.who.int/bitstream/handle/10665/134588/WHO_RHR_14.23_eng.pdf?sequence=1 (Accessed November 11, 2024).

[21] WHO. Framework for Action Strengthening Quality of Midwifery Education for Universal Health Coverage 2030 (2019). Available at: http://www.midwife.org.tw/upfiles/newsfiles/9789241515849-eng.pdf (Accessed November 11, 2024).

[22] Brady S Bogossian F Gibbons KS. Defining woman-centred care: a concept analysis. Midwifery. (2024) 131(103954 December 2023):1–9. 10.1016/j.midw.2024.103954 38364459

[23] Sposato MF Miller WR. Concept analysis of woman-centered care: implications for postpartum care. MCN Am J Matern Child Nurs. (2024) 49(6):314–23. 10.1097/NMC.0000000000001045 39012337

[24] Watkins V Nagle C Kent B Street M Hutchinson AM. Labouring together: women’s experiences of ‘getting the care that I want and need’ in maternity care. Midwifery. (2022) 113:103420. 10.1016/j.midw.2022.103420 35849913

[25] Fontein-Kuipers Y de Groot R van Beeck E van Hooft S van Staa AL. Dutch midwives’ views on and experiences with woman-centred care — a Q-methodology study. Women Birth. (2019) 32(6):e567–75. 10.1016/j.wombi.2019.01.003 30685135

[26] Maputle MS Donavon H. Woman-centred care in childbirth: a concept analysis (part 1). Curationis. (2013) 36(1):E1–8. 10.4102/curationis.v36i1.49 23718832

[27] Fahy K. What is woman-centred care and why does it matter? Women Birth. (2012) 25(4):149–51. 10.1016/j.wombi.2012.10.005 23177665

[28] Leap N. Woman-centred or women-centred care: does it matter? Br J Midwifery. (2009) 17(1):12–6. 10.12968/bjom.2009.17.1.37646

[29] Downe S Finlayson K Oladapo OT Bonet M Metin Gülmezoglu A. What matters to women during childbirth: a systematic qualitative review. PLoS One. (2018) 13(4):e0194906. 10.1371/journal.pone.0194906 29664907 PMC5903648

[30] Oladapo OT Tunçalp O Bonet M Lawrie TA Portela A Downe S WHO model of intrapartum care for a positive childbirth experience: transforming care of women and babies for improved health and wellbeing. BJOG. (2018) 125(8):918–22. 10.1111/1471-0528.15237 29637727 PMC6033015

[31] Perriman N Davis DL Ferguson S. What women value in the midwifery continuity of care model: a systematic review with meta-synthesis. Midwifery. (2018) 62(February):220–29. 10.1016/j.midw.2018.04.011 29723790

[32] Olza I Leahy-Warren P Benyamini Y Kazmierczak M Karlsdottir SI Spyridou A Women’s psychological experiences of physiological childbirth: a meta-synthesis. BMJ Open. (2018) 8(10):1–11. 10.1136/bmjopen-2017-020347 PMC6196808 30341110

[33] Leinweber J Fontein-Kuipers Y Thomson G Karlsdottir SI Nilsson C Ekström-Bergström A Developing a woman-centered, inclusive definition of traumatic childbirth experiences: a discussion paper. Birth. (2022) 49(4):687–96. 10.1111/birt.12634 35403241

[34] Sandall J Fernandez Turienzo C Devane D Soltani H Gillespie P Gates S Midwife continuity of care models versus other models of care for childbearing women. Cochrane Database Syst Rev. (2024) 4(4):CD004667. 10.1002/14651858.CD004667.pub6 38597126 PMC11005019

[35] O’Reilly E Buchanan K Bayes S. Emotional safety in maternity care: an evolutionary concept analysis. Midwifery. (2025) 140(October 2024):104220. 10.1016/j.midw.2024.104220 39514941

[36] Bohren MA Mehrtash H Fawole B Maung TM Balde MD Maya E How women are treated during facility-based childbirth in four countries: a cross-sectional study with labour observations and community-based surveys. Lancet. (2019) 394(10210):1750–63. 10.1016/S0140-6736(19)31992-0 31604660 PMC6853169

[37] Shorey S Ng ED. Midwives’ perceptions of and experiences with normal physiologic birth: a qualitative systematic review. Birth. (2023) 50(4):749–63. 10.1111/birt.12763 37712184

[38] Crepinsek M Bell R Graham I Coutts R. Towards a conceptualisation of woman centred care — a global review of professional standards. Women Birth. (2022) 35(1):31–7. 10.1016/j.wombi.2021.02.005 33676876

[39] Bull C Teede H Carrandi L Rigney A Cusack S Callander E. Evaluating the development, woman-centricity and psychometric properties of maternity patient-reported outcome measures (PROMs) and patient-reported experience measures (PREMs): a systematic review protocol. BMJ Open. (2022) 12(2):1–9. 10.1136/bmjopen-2021-058952 PMC8845328 35144957

[40] Fontein-Kuipers Y de Groot R van Staa A. Woman-centered care 2.0: bringing the concept into focus. Eur J Midwifery. (2018) 2:5. 10.18332/ejm/91492 33537566 PMC7846029

[41] International Confederation of Midwives. ICM Definitions. Philosophy and Model of Midwifery Care (2014). Available at: https://www.internationalmidwives.org/our-work/policy-and-practice/icm-definitions.html (Accessed February 01, 2025).

[42] Homer CSE. Models of maternity care: evidence for midwifery continuity of care. Med J Aust. (2016) 205(8):370–74. 10.5694/mja16.00844 27736625

[43] Renfrew MJ McFadden A Bastos MH Campbell J Channon AA Cheung NF Midwifery and quality care: findings from a new evidence-informed framework for maternal and newborn care. Lancet. (2014) 384(9948):1129–45. 10.1016/S0140-6736(14)60789-3 24965816

[44] Hunter B Berg M Lundgren I Ólafsdóttir ÓÁ Kirkham M. Relationships: the hidden threads in the tapestry of maternity care. Midwifery. (2008) 24(2):132–37. 10.1016/j.midw.2008.02.003 18378051

[45] Leap N Dodwell NCT Newburn M. Working with pain in labour. Res Dig Natl Childbirth Trust. (2010) 49(12):22–6.

[46] Leap N Sandall J Buckland S Huber U. Journey to confidence: women’s experiences of pain in labour and relational continuity of care. J Midwifery Womens Health. (2010) 55(3):234–42. 10.1016/j.jmwh.2010.02.001 20434083

[47] Bradfield Z Hauck Y Kelly M Duggan R. ‘It’s what midwifery is all about’: western Australian Midwives’ experiences of being ‘with woman’ during labour and birth in the known midwife model. BMC Pregnancy Childbirth. (2019) 19(1):29. 10.1186/s12884-018-2144-z 30642287 PMC6332887

[48] Skeide A. Enacting homebirth bodies: midwifery techniques in Germany. Cult Med Psychiatry. (2019) 43(2):236–55. 10.1007/s11013-018-9613-8 30484002

[49] O’Brien D Butler MM Casey M. The importance of nurturing trusting relationships to embed shared decision-making during pregnancy and childbirth. Midwifery. (2021) 98(June 2020):102987. 10.1016/j.midw.2021.102987 33761433

[50] Naughton S Baldwin A Harvey C Capper T. The midwifery capabilities theory: how midwives enact woman-centered care to address systemic inequity. Birth. (2024) 00:1–10. 10.1111/birt.12866 PMC12434215 39297756

[51] Feeley C. Skilled Heartfelt Midwifery Practice: Safe, Relational Care for Alternative Physiological Births. 1st ed. 20. Cham: Springer International Publishing (2023). 10.1007/978-3-031-43643-7

[52] Stone NI Thomson G Tegethoff D. Tailoring midwifery care to women’s needs in early labour: the cultivation of relational care in free-standing birth centres. Midwifery. (2025) 140(October 2024):104202. 10.1016/j.midw.2024.104202 39395312

[53] Bradford BF Wilson AN Portela A McConville F Turienzo CF Homer CSE. Midwifery continuity of care: a scoping review of where, how, by whom and for whom? PLOS Global Public Health (2022) 2(10):e0000935. 10.1371/journal.pgph.0000935 36962588 PMC10021789

[54] Jonge Ad Dahlen H Downe S. ‘Watchful attendance’ during labour and birth. Sex Reprod Healthc. (2021) 28:100617. 10.1016/j.srhc.2021.100617 33774268

[55] Healy S Humphreys E Kennedy C. A qualitative exploration of how midwives ‘ and obstetricians ’ perception of risk affects care practices for low-risk women and normal. Women Birth. (2017) 30(5):367–75. 10.1016/j.wombi.2017.02.005 28279637

[56] Downe S Agius JC Balaam M-C Frith L. Understanding childbirth as a complex salutogenic phenomenon: the EU COST BIRTH action special collection. PLoS One. (2020) 15(8):e0236722. 10.1371/journal.pone.0236722 32756586 PMC7406045

[57] Newnham E Kirkham M. Beyond autonomy: care ethics for midwifery and the humanization of birth. Nurs Ethics. (2019) 26(7–8):2147–57. 10.1177/0969733018819119 30638112

[58] Thompson FE. Moving from codes of ethics to ethical relationships for midwifery practice. Nurs Ethics. (2002) 9(5):522–36. 10.1191/0969733002ne542oa 12238748

[59] MacLellan J. Claiming an ethic of care for midwifery. Nurs Ethics. (2014) 21(7):803–11. 10.1177/0969733014534878 24917265

[60] van der Waal R van Nistelrooij I. Reimagining relationality for reproductive care: understanding obstetric violence as ”separation”. Nurs Ethics. (2022) 29(5):1186–97. 10.1177/09697330211051000 35100071 PMC9445229

[61] Buchanan K Newnham E Ireson D Davison C Bayes S. Does midwifery-led care demonstrate care ethics: a template analysis. Nurs Ethics. (2022) 29(1):245–57. 10.1177/09697330211008638 34396811

[62] Pols J. Reinventing the Good Life: An Empirical Contribution to the Philosophy of Care. London: UCL Press (2023).

[63] Cohn S Driessen A Borgstrom E. Human and person when life is Fragile: new relationships and inherent ambivalences in the care of dying patients. Sci Technol Hum Values. (2023) 0(0). 10.1177/01622439231155647

[64] Vogel E. Tinkering with relations: veterinary work in Dutch farm animal care. In: Tallberg L Hamilton L, editors. The Oxford Handbook of Animal Organization Studies. Oxford: Oxford University Press (2022). p. 288–99. 10.1093/oxfordhb/9780192848185.013.19

[65] Mol A Moser I Pols J. Care in Practice. on Tinkering in Clinics, Homes and Farms. VerKörperungen 8. Bielefeld: Transcript Verlag (2010).

[66] Law J. Actor network theory and material semiotics. In: Turner BS, editor. The New Blackwell Companion to Social Theory. Hoboken, New Jersey: Wiley-Blackwell (2009). p. 141–58. 10.1002/9781444304992.ch7

[67] Pols J. Radical relationality. Epistemology in care and care ethics for research. In: Olthuis G Kohlen H Heier J, editors. Moral Boundaries Redrawn the Significance of Joan Tronto’s Argument for Political Theory, Ethics of Care. Leuven: Peeters (2014). p. 175–94.

[68] Mol A. The Body Multiple: Ontology in Medical Practice. Durham, NC: Duke University Press (2002).

[69] Mol A. The Logic of Care: Health and the Problem of Patient Choice. London: Routledge (2008).

[70] Mol A Hardon A. Caring. In: Bowen JR Dodier N Duyvendak JW Hardon A, editors. Pragmatic Inquiry: Critical Concepts for Social Sciences. Abingdon, Oxon: Routledge (2021). p. 185–204.

[71] Akrich M Pasveer B. Embodiment and disembodiment in childbirth narratives. Body Soc. (2004) 10(2–3):63–84. 10.1177/1357034X04042935

[72] Despret V. The body we care for: figures of anthropo-zoo-genesis. Body Soc. (2004) 10(2–3):111–34. 10.1177/1357034X04042938

[73] Abrahamsson S. An actor network analysis of constipation and agency: shit happens. Subjectivity. (2014) 7(2):111–30. 10.1057/sub.2014.5

[74] Hennion A. Music lovers: taste as performance. Theory Cult Soc. (2001) 18(5):1–22. 10.1177/02632760122051940

[75] Cussins C. Ontological choreography: agency through objectification in infertility clinics. Soc Stud Sci. (1996) 26(3):575–610. 10.1177/030631296026003004

[76] Downe S Stone NI. Midwives and midwifery. The need for courage to reclaim vocation for respectful care. In: Pickles C Herring J, editors. Childbirth, Vulnerability and Law. Exploring Issues of Violence and Control. Abingdon, UK: Routledge (2019). p. 88–110.

[77] Skeide A. Witnessing as an embodied practice in German midwifery care. In: Krause F Boldt J, editors. Care in Healthcare: Reflections on Theory and Practice. Cham: Palgrave Macmillan (2018). p. 191–209. 10.1007/978-3-319-61291-1_10 31314348

[78] Skeide A. Experiences as actors: labor pains in childbirth care in Germany. Med Anthropol. (2021) 40(5):446–57. 10.1080/01459740.2020.1860963 33400594

[79] Skeide A. Music to my ears: a material-semiotic analysis of fetal heart sounds in midwifery prenatal care. Sci Techno Hum Values. (2021) 47(3):517–43. 10.1177/01622439211005176

[80] Braun V Clarke V. Reflecting on reflexive thematic analysis. Qual Res Sport Exerc Health. (2019) 11(4):589–97. 10.1080/2159676X.2019.1628806

[81] Braun V Clarke V. (Mis)Conceptualising themes, thematic analysis, and other problems with Fugard and Potts’ (2015) sample-size tool for thematic analysis. Int J Soc Res Methodol. (2016) 19(6):739–43. 10.1080/13645579.2016.1195588

[82] Braun V Clarke V. Using thematic analysis in psychology. Qual Res Psychol. (2006) 3(2):77–101. 10.1191/1478088706qp063oa

[83] Blumer H. What is wrong with social theory? Am Sociol Rev. (1954) 19(1):3–10. 10.2307/2088165

[84] QUAG (Gesellschaft für Qualität in der außerklinischen Geburtshilfe). Geburtenzahlen in Deutschland (2024) Available at: https://www.quag.de/quag/geburtenzahlen.htm (Accessed November 25, 2024).

[85] Davis-Floyd RE. The technocratic body: American childbirth as cultural expression. Soc Sci Med. (1994) 38(8):1125–40. 10.1016/0277-9536(94)90228-3 8042048

[86] Rothman BK. In Labor: Women and Power in the Birthplace. New York: Norton (1982).

[87] Martin E. The Woman in the Body. A Cultural Analysis of Reproduction. Boston: Beacon Press (1987).

[88] Lupton D Schmied V. Splitting bodies/selves: women’s concepts of embodiment at the moment of birth. Sociol Health Illn. (2013) 35(6):828–41. 10.1111/j.1467-9566.2012.01532.x 23094983

[89] Young I. Pregnant embodiment. In: Welton D, editor. Body and Flesh: A Philosophical Reader. Malden, Mass: Blackwell (1998). p. 274–85.

[90] Pols J. Enacting appreciations: beyond the patient perspective. Int J Health Care Philos Policy. (2005) 13(3):203–21. 10.1007/s10728-005-6448-6 16223211

[91] Pasveer B Akrich M. Obstetrical trajectories. On training women/bodies for (home)Birth. In: De Vries R Bemoit C van Teijlingen E Wrede S, editors. Birth by Design. Pregnancy, Maternity Care and Midwifery in North America and Europe. New York: Routledge (2001). p. 229–42.

[92] Scamell M Alaszewski A. Fateful moments and the categorisation of risk: midwifery practice and the ever-narrowing window of normality during childbirth. Health Risk Soc. (2012) 14(2):207–21. 10.1080/13698575.2012.661041

[93] Moreira TE. Self, agency and the surgical collective: detachment. Sociol Health Illn. (2004) 26(1):32–49. 10.1111/j.1467-9566.2004.00377.x 15027989

[94] Candea M. I fell in love with carlos the meerkat: engagement and detachment in human-animal relations. Am Ethnol. (2010) 37(2):241–58. 10.1111/j.1548-1425.2010.01253.x

[95] Cohen Shabot S. Making loud bodies “feminine”: a feminist-phenomenological analysis of obstetric violence. Hum Stud. (2016) 39(2):231–47. 10.1007/s10746-015-9369-x

[96] Walsh DJ. Childbirth embodiment: problematic aspects of current understandings. Sociol Health Illn. (2010) 32(3):486–501. 10.1111/j.1467-9566.2009.01207.x 20003040

[97] Puig De La Bellacasa M. Matters of Care. Matters of Care. Speculative Ethics in More Than Human Worlds. Minneapolis: University of Minnesota Press (2017).

[98] Cohn S. From health behaviours to health practices: an Introduction. Soc Health Illn. (2014) 36(2):157–62. 10.1111/1467-9566.12140 24528302

[99] Hoyert DL. Maternal mortality rates in the United States, 2022. (2024). Available at: https://www.cdc.gov/nchs/data/hestat/maternal-mortality/2022/maternal-mortality-rates-2022.pdf (Accessed January 05, 2025). 39946528

[100] Geller SE Koch AR Garland CE MacDonald EJ Storey F Lawton B. A global view of severe maternal morbidity: moving beyond maternal mortality. Reprod Health. (2018) 15(Suppl 1):98. 10.1186/s12978-018-0527-2 29945657 PMC6019990

[101] Miller S Abalos E Chamillard M Ciapponi A Colaci D Comandé D Beyond too little, too late and too much, too soon: a pathway towards evidence-based, respectful maternity care worldwide. Lancet. (2016) 388(10056):2176–92. 10.1016/S0140-6736(16)31472-6 27642019

